# Supplementary material for: Impact of Insoluble Separation Layer Mechanical Properties on Disintegration and Dissolution Kinetics of Multilayer Tablets
Source: Pharmaceutics. 2020 May 29;12(6):495. doi: 10.3390/pharmaceutics12060495 (PMC7356680; doi:10.3390/pharmaceutics12060495)
Supplement: Supplementary file 1 [file pharmaceutics-12-00495-s001.pdf]

pharmaceutics

MDPI

Supplementary Materials: Impact of Insoluble Separation Layer Mechanical Properties on Disintegration and Dissolution Kinetics of Multilayer Tablets

Reiji Yokoyama, Go Kimura, Jörg Huwyler, Ken-ichi Hosoya and Maxim Puchkov *

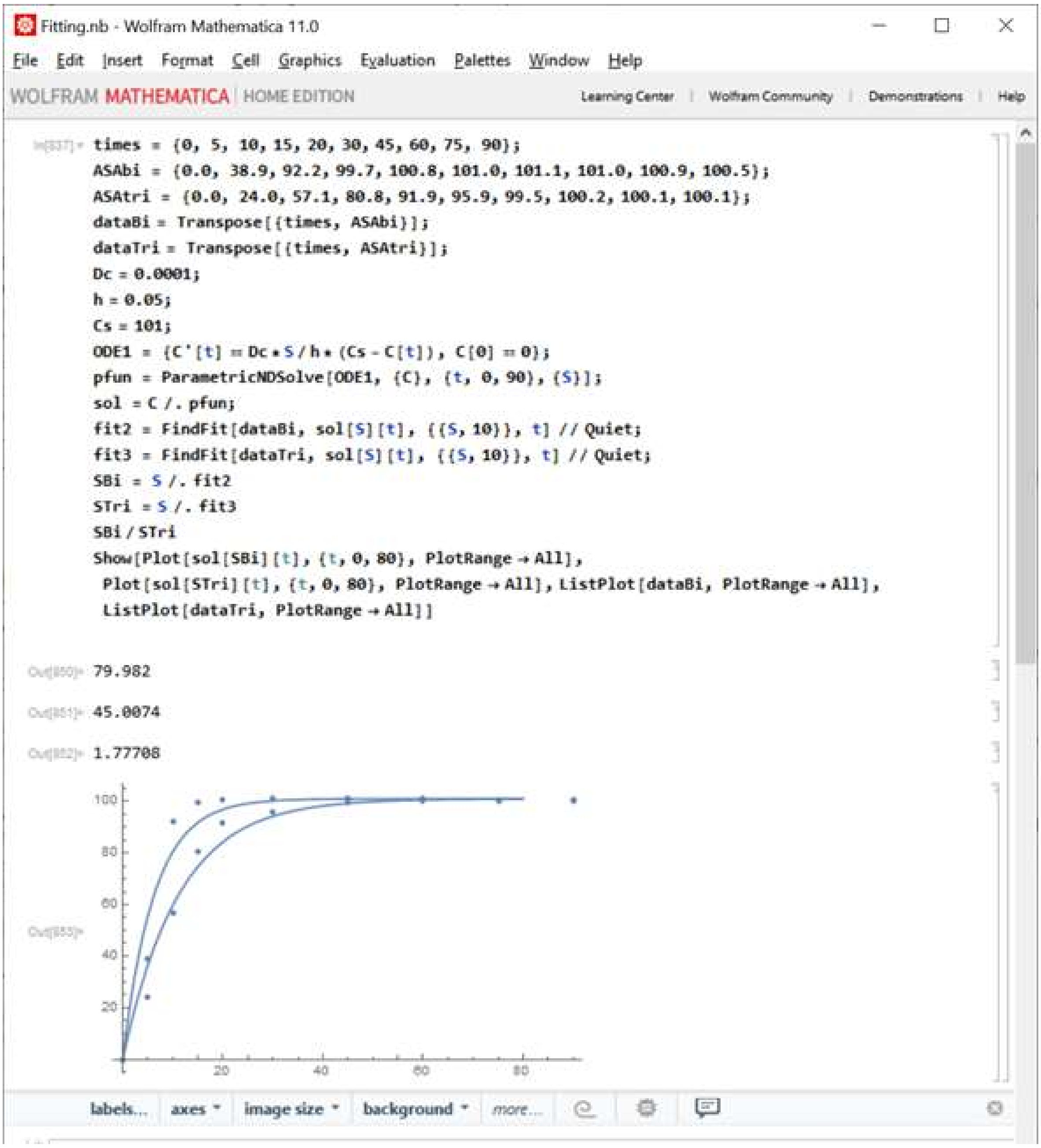

Figure S1. Screenshot of Wolfram Mathematica.
